# Crystal structure of Type IX secretion system PorE C-terminal domain from *Porphyromonas gingivalis* in complex with a peptidoglycan fragment

**DOI:** 10.1038/s41598-020-64115-z

**Published:** 2020-04-30

**Authors:** Nhung Thi Trang Trinh, Hieu Quang Tran, Quyen Van Dong, Christian Cambillau, Alain Roussel, Philippe Leone

**Affiliations:** 1 Architecture et Fonction des Macromolécules Biologiques, Aix-Marseille Université, UMR 7257, 163 Avenue de Luminy, Case 932, 13009 Marseille, France; 2 Architecture et Fonction des Macromolécules Biologiques, Centre National de la Recherche Scientifique, UMR 7257, 163 Avenue de Luminy, Case 932, 13009 Marseille, France; 3 Institute of Biotechnology, Vietnam Academy of Science and Technology. 18 Hoang Quoc Viet, Ha Noi, Vietnam; 4 University of Science and Technology of Hanoi, Vietnam Academy of Science and Technology. 18 Hoang Quoc Viet, Ha Noi, Vietnam

**Keywords:** X-ray crystallography, Bacterial secretion, Bacterial pathogenesis

## Abstract

*Porphyromonas gingivalis*, the major human pathogen associated to periodontal diseases, utilizes the *Bacteroidetes*-specific type IX secretion system (T9SS) to export virulence factors. PorE is a periplasmic multi-domain lipoprotein associated to the outer membrane that was recently identified as essential for T9SS function. Little is known on T9SS at the structural level, and in particular its interaction with peptidoglycan. This prompted us to carry out structural studies on PorE full length as well as on its four isolated domains. Here we report the crystal structure of the C-terminal OmpA_C-like putative peptidoglycan-binding domain at 1.55 Å resolution. An electron density volume was identified in the protein cleft, making it possible to build a naturally-occurring peptidoglycan fragment. This result suggests that PorE interacts with peptidoglycan and that PorE could anchor T9SS to the cell wall.

## Introduction

Periodontal disease, or periodontitis, is a chronic inflammatory disease that destroys periodontal tissue and alveolar bone, representing a major public health concern. The Gram-negative anaerobic bacterium *Porphyromonas gingivalis* is a pathogen highly associated with periodontal diseases. *P. gingivalis* can locally invade periodontal tissues, disrupt the tooth-supporting structure and evade the host defense mechanisms. Importantly, several studies have revealed that there are cause-and-effect relationships between periodontal diseases and rheumatoid arthritis, heart disease, diabetes, Alzheimer and other systemic diseases^1–7^. Tissue damages caused by *P. gingivalis* infection are mainly induced by a cocktail of specialized toxin proteins secreted by the bacterium, the gingipains^8^. Several studies have demonstrated that *P. gingivalis* employs the Type IX secretion system (T9SS) to translocate gingipains across the outer membrane (OM)^9,10^. Therefore, it is important to gather structure/function data on T9SS to fight *P. gingivalis* virulence.

T9SS is present in the *Fibrobacteres-Chlorobi-Bacteroidetes* superphylum (CFB group). It has been mainly studied in the oral pathogen *P. gingivalis* and in the gliding bacterium *F. johnsoniae*. The *P. gingivalis* T9SS recruits and delivers virulence factors, such as gingipains, to attack to the host periodontal cells, while the *F. johnsoniae* T9SS secretes adhesins that promote gliding motility. Effectors secreted by the T9SS use a two-step mechanism. Substrate proteins have cleavable N-terminal signal peptides that target them first to the Sec translocon for export across the inner membrane (IM). They also harbour a conserved carboxy-terminal domain (CTD) comprising approximately 80 residues that is necessary for recruitment to and transport across the OM by the T9SS^11^. T9SS is composed of at least 14 different proteins. Worth noticing, the location of the *P. gingivalis* T9SS subunits have been determined within the cell envelope: two are IM embedded proteins (PorL and PorM), six are OM embedded proteins (PG0189, PorT, PorP, Sov, PorV and PorQ), five are OM-associated proteins (PorE, PorK, PorW, PorU and PorZ) and one is a periplasmic protein (PorN)^10,12–19^. However, few is known at the structural level. It has been shown by cryo-electron microscopy (cryo-EM) that PorK and PorN polymerize to form a large ring-shaped complex^20^. Recently, the crystal structures of the periplasmic domain of PorM from *P. gingivalis* and its homologue GldM from *F. johnsoniae* have been solved^21^, proposing a schematic model of their function. Finally, the cryo-EM structure of the SprA protein (Sov homolog in *F. johnsoniae*) was solved in complex with its partner proteins, PorV, PPI (lipoprotein peptidyl-prolyl cis-trans isomerase) and Plug protein^22^. The structure showed that SprA folds as a large 36-strand single polypeptide transmembrane β−barrel. This β−barrel is gated by a plug and displays a lateral opening that might be important for effector translocation.

In 2016, a new T9SS subunit was identified, PorE, initially named PG1058 (Uniprot code F5HF80)^17,23^. PorE locates in the periplasm and is anchored to the inner leaflet of the OM through its lipid modification^17^. In the same study, it was shown that PorE is essential for T9SS function. Indeed, the *porE* mutant in *P. gingivalis* displays phenotypes characteristic of non-functional T9SS: defect in colonial pigmentation, defect in surface-associated proteolytic activity and absence of a visible electron dense surface layer (EDSL) containing gingipains and other substrate proteins by cryo-EM^17^. Sequence analysis and computer modelling indicated that PorE could be divided into four domains: i) a tetratricopeptide repeat domain (TPR, residues 25–149); ii) a β–propeller domain (WD40, residues 167–435); iii) a carboxypeptidase regulatory domain-like fold (CRD, residues 441–527) and iv) an OmpA_C-like putative peptidoglycan-binding domain (residues 534–668). The TPR and WD40 domains present at the N-terminus of PorE are structural scaffolds often involved in protein-protein interactions^24,25^, suggesting that PorE may have a role as an essential scaffold linking the periplasmic and OM components of the T9SS. The OmpA-like C-terminus domain is similar to peptidoglycan binding proteins in other bacteria, suggesting that PorE N- to C-terminus could establish a bridge between the OM and the peptidoglycan.

Peptidoglycan (PG) is the major component of the bacterial cell wall and shapes the cell by giving structural strength. PG is a polymer consisting of repeating disaccharide units of N -acetylglucosoamine (NAG) and N-acetylmuramic acid (NAM). A PG strand is cross-linked to the other neighbouring PG strands through a pentapeptide stem, which is appended to the NAM moiety. In gram-negative bacteria, _L_-Ala-γ-_D_-Glu-*meso*-DAP-_D_-Ala-_D_-Ala is the common pentapeptide, in which DAP represents diaminopimelate, a unique bacterial amino acid^26,27^. It was shown only recently that *P. gingivalis* peptidoglycan contains *meso*-DAP and not the stereochemical variant LL-DAP as believed for a long time^28,29^. PG is located between the IM and OM of gram-negative bacteria. The OmpA family proteins have been proposed to interact noncovalently with PG^30^. There are three different groups in the OmpA family proteins: OM proteins (OmpA, NmRmpM, CadF, OprF, and others), PG-associated lipoproteins (Pal, OprL, and others), and proteins associated with large machineries (MotB/MotY, ExeA, TagL, which are associated with the flagellar rotor, the Type II and Type VI secretion systems, respectively)^31–34^. While OM proteins and PG-associated lipoproteins connect the OM with the PG network and hence stabilize the OM, proteins associated with multiprotein machineries usually help to proper position and immobilize the apparatus^35^.

In this study we focused on the *P. gingivalis* PorE T9SS-associated lipoprotein. The full-length mature PorE protein and its four truncated domains were cloned, produced and one domain was crystallized. The crystal structure of the OmpA_C-like putative peptidoglycan-binding domain was solved in complex with a part of the naturally-occurring peptidoglycan peptide stem, bound to each monomer of the tetrameric arrangement in the asymmetric unit.

## Results

### Structure determination of the PorE OmpA_C-like domain

We attempted to produce the full length PorE as well as the four isolated domains defined by Heath *et al*.^17^. The full-length protein and the β–propeller (WD40) domain were insoluble. The production yield of the tetratricopeptide repeat (TPR) domain was very low. By contrast, the carboxypeptidase regulatory domain-like fold (CRD) and the OmpA_C-like putative peptidoglycan-binding constructs were produced in high yield and were soluble.

Crystallization trials were performed with these two domains and crystals were obtained only for the C-terminal OmpA_C-like domain. The crystals belong to the space group C2 with unit-cell parameters a = 208.0 Å, b = 52.2 Å, c = 66.5 Å and α = γ = 90°, β = 108.4°. The structure of the PorE OmpA_C-like domain was solved by single-wavelength anomalous diffraction (SAD) at 1.55 Å resolution (Table 1) using selenomethionine containing protein. The Matthews coefficient calculated with four molecules in the asymmetric unit is 2.38 Å^3^ Da^−1^, corresponding to an estimated solvent content of 48.41%. The four molecules present in the asymmetric unit form a tightly packed tetramer that is predicted to be stable in solution by the PDBe PISA server (calculated buried area: 11560 Å^2^). However, this oligomerization state results from a crystallization artefact as Size-Exclusion Chromatography Multi-Angle Light Scattering (SEC-MALS) analysis indicated that the protein in solution is monomeric with a molecular weight of 17.7 kDa (Fig. S1). The superimposition of the four monomers shows rmsd (root mean square deviation) comprised between 0.3 and 0.6 Å indicating that the four molecules are almost identical.

**Table 1:** Data collection and refinement statistics of the PorE OmpA_C-like domain.

**Data collection**	
PDB accession code	6TOP
Space group	C 2
a, b, c (Å)	208.0, 52.2, 66.56
α, β, γ (°)	90, 108.4, 90
Resolution (Å)*	49.35–1.55 (1.59–1.55)
Unique reflections*	96340 (5637)
Redundancy*	22 (14)
Completeness (%)*	97.6 (77.6)
I/σ*	19.7 (1.5)
R_meas_ (%)*	9.0 (185)
CC1/2	0.999 (0.85)
Mosaicity (°)	0.075
**Refinement and model quality**	
Resolution (Å)*	49.35–1.55 (1.56–1.55)
Reflections*	96279 (1813)
R_fac_/R_free_ (%)	19.0/21.5
N° atoms:	
protein/ligand/ion/water	4825/121/4/756
B-factors (Å^2^):	
protein/ligand/ion/water	34.6/50.3/36.2/45.0
Rmsd: bond (Å)/angle (°)	0.008/0.95
Ramachandran (%):	
most favoured	98.83
allowed regions	1.17
outliers	0

*Values in parentheses are for the highest-resolution shell.

### Overall structure and peptidoglycan binding site

**Figure 1:**
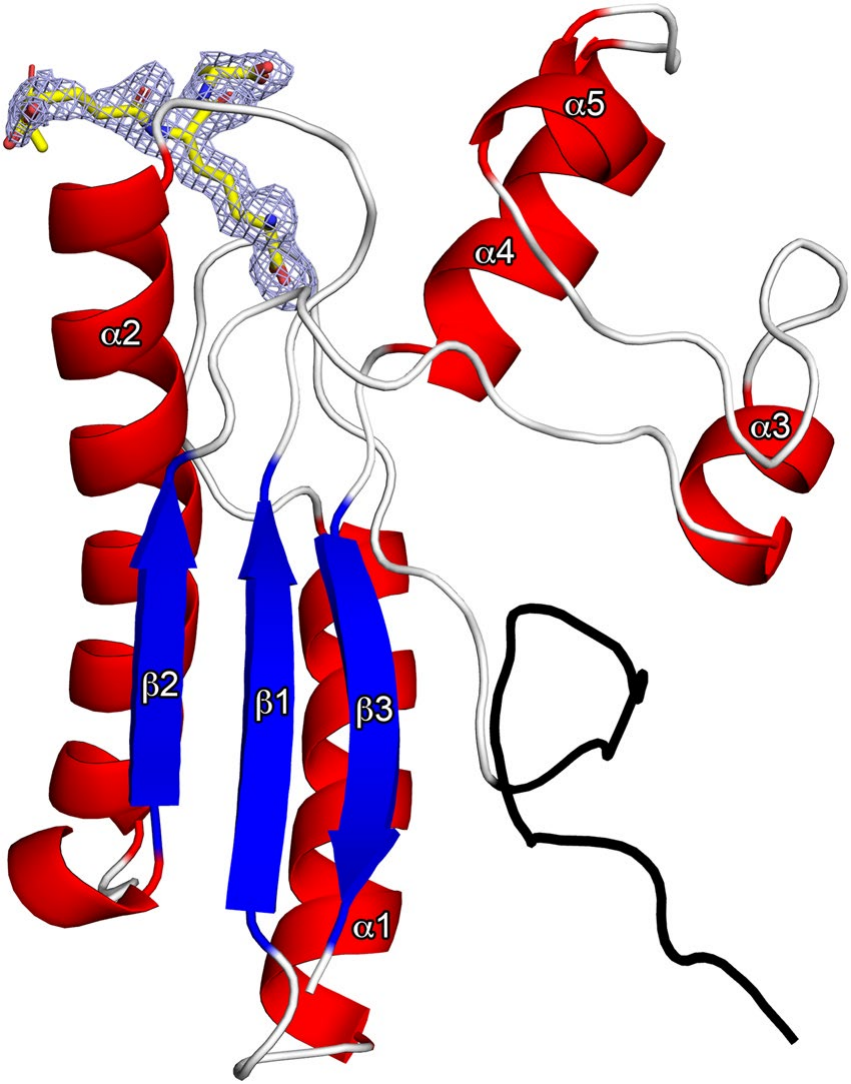
Overall structure of the PorE OmpA_C-like domain in complex with a peptidoglycan fragment. The PorE OmpA_C-like domain is shown as a ribbon diagram with red α-helices and blue β-sheets; the N- and C-termini are labelled. The N-terminal residues corresponding to the end of the His_6_ tag and the TEV cleavage site encoded in the expression vector are colored in black. The peptidoglycan fragment (_L_-Ala-γ-_D_-Glu-*meso*-DAP-_D_-Ala) is displayed in yellow stick format; its omit difference electron density map (blue) is contoured at 3σ. The figure was prepared using PyMOL (version 1.20, https://pymol.org).

The structure of the OmpA_C-like domain of PorE (residues 534 to 668) consists of a three-stranded β-sheet (β1-3) and five α-helices (α1–5) with connectivity α1β1α2β2α3α4α5β3 (Fig. 1). Helices α1 and α2 pack against the β–sheet on one side while helices α3, α4 and α5 form an insertion between the β2 and β3 strands. Within the SCOP classification, domains of the OmpA-like superfamily comprise a four-stranded β-sheet. In the PorE OmpA-like domain structure, the first β strand is missing and is fortunately replaced by extra residues encoded in the expression vector. Indeed, the −19 to −1 first residues present in the electron density map do not belong to the sequence of PorE as they correspond to the end of the His_6_ tag and the TEV cleavage site (colored black in the structure).

To note, the structure of the OmpA_C-like domain features a relatively large bulge containing the two small α-helices, α3 and α4, which are not connected by a disulfide bond as it is the case in some other well-known peptidoglycan-associated proteins, for examples, *E. coli* OmpA^36^ or *Neisseria meningitides* RmpM^37^.

**Figure 2:**
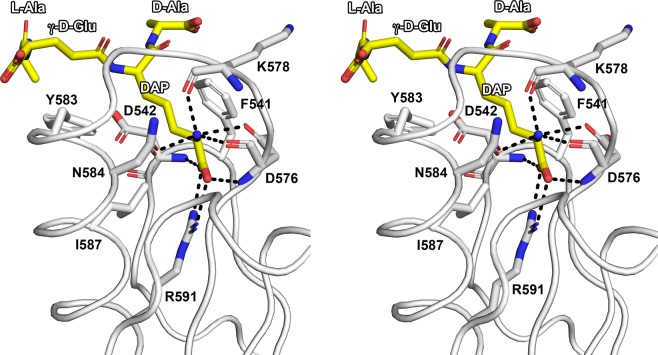
Stereo view of the interactions network between the PorE OmpA_C-like domain (white) and the peptidoglycan fragment (yellow). PorE and peptidoglycan residues are labelled in black and white, respectively. Hydrogen bonds are displayed as black dotted lines. Nitrogen and oxygen atoms are displayed in blue and red, respectively. The orientation is the same as in Fig. 1. The figure was prepared using PyMOL (version 1.20, https://pymol.org).

The surface of the OmpA_C-like domain displays a narrow and deep cavity in which extra electron density was readily visible allowing the building of a naturally-occurring fragment of PG containing four residues of the stem peptide, _L_-Ala-γ-_D_-Glu-*meso*-DAP-_D_-Ala (Fig. 1). This PG fragment interacts strongly with the protein, principally through the DAP residue, as it remained bound after the whole purification and crystallization process. Indeed, the DAP residue is deeply embedded into the cavity; the carboxylate is engaged in a bidentate salt bridge with the guanidinium NH1 and NH2 of Arg591, and forms hydrogen bonds to the main-chain amide protons of Asp542 and Asp576 (Fig. 2). The NBW of DAP is hydrogen bonded to the main-chain amide proton and the OD1 of Asp576, to the main-chain amide proton of Lys 578, and to the OD1 of Asn584. Furthermore, a hydrophobic contacts network further stabilizes the interaction: Phe541 interacts with DAP and _L_-Ala, Tyr583 interacts with γ-_D_-Glu and _D_-Ala, and Ile587 interacts with DAP (Fig. 2).

**Figure 3:**
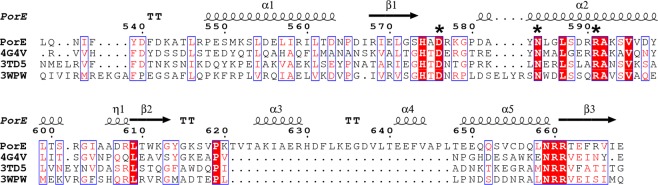
Sequence alignment of the PorE OmpA_C-like domain with other representative OmpA family proteins. Secondary structure elements and numeration from the OmpA_C-like domain of PorE structure are displayed above the alignment. Strictly conserved residues are in red blocks; the three residues of the PorE OmpA_C-like domain whose side chains are involved in hydrogen bonds with the peptidoglycan fragment are indicated by an asterisk. 4G4V: peptidoglycan-associated lipoprotein (PAL) from *Acinetobacter baumannii*; 3TD5, OmpA family protein from *Acinetobacter baumannii*; 3WPW, flagellar stator coupled protein PomB from *Vibrio alginolyticus*. Amino acid sequences were aligned using T-Coffee (version 11.00.d625267, http://www.tcoffee.org)^50^, and the figure was prepared using ESPript (version 3.0, http://espript.ibcp.fr/)^51^.

**Figure 4:**
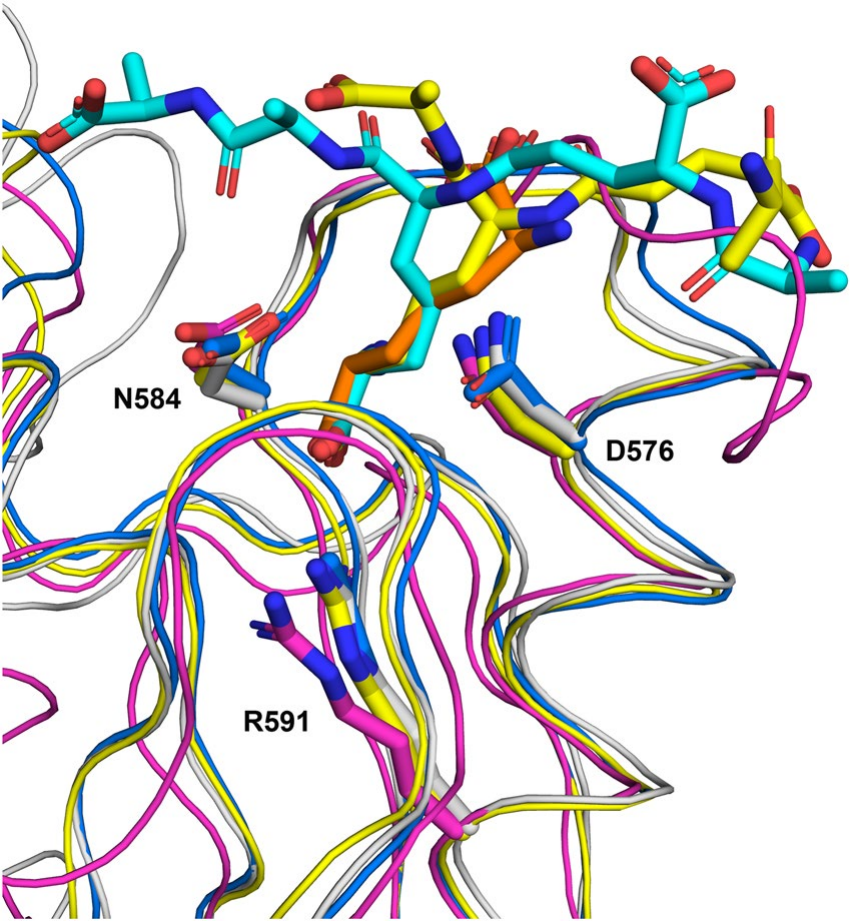
Superimposition of the PorE OmpA_C-like domain structure with other representative OmpA family proteins (same as in Fig. 3). Structures of the proteins are displayed as a ribbon diagram with worm format; the peptidoglycan (PG) fragments as well as the three conserved residues whose side chain is involved in hydrogen bonds with the PG fragment are displayed in stick format, with nitrogen and oxygen atoms displayed in blue and red, respectively. The protein chains and their bound PG fragments are respectively shown in white and grey (PorE OmpA_C-like domain), yellow and orange (*A. baumannii* PAL, 4G4V), and blue and cyan (*A. baumannii* OmpA, 3TD5). *V. alginolyticus* PomB (3WPW), that contains no PG fragment, is shown in magenta. For clarity purpose, only the PorE OmpA_C-like domain residues are labelled, and the orientation is rotated 90° from Figs. 1 and 2 along the vertical axis. The figure was prepared using PyMOL (version 1.20, https://pymol.org).

**Figure 5:**
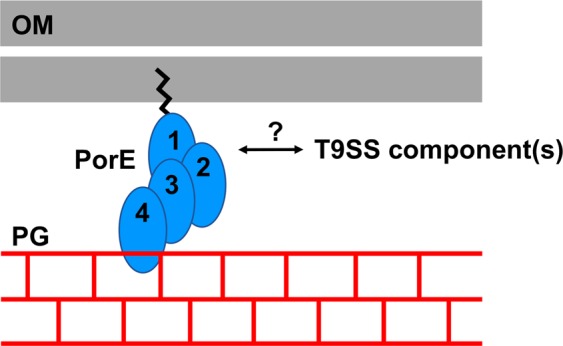
Schematic representation of the putative role of PorE in anchoring T9SS to the bacterial cell wall. PorE is a lipoprotein composed of four domains (1: tetratricopeptide repeat domain (TPR), 2: β–propeller domain (WD40), 3: carboxypeptidase regulatory domain-like fold (CRD), and 4: OmpA_C-like putative peptidoglycan-binding domain). Therefore, PorE is anchored to the inner leaflet of the OM through its N-terminal acylation, and could interact with the peptidoglycan network through the C-terminal OmpA_C-like domain. Interaction with (a) putative T9SS component(s) could be mediated by the TPR and WD40 domains that are structural scaffolds often involved in protein-protein interactions. The figure was prepared using MICROSOFT PowerPoint (version 16.16.20).

The three residues whose side chains are involved in hydrogen bonds with PG (Asp576, Asn584 and Arg591) are conserved among OmpA family proteins, such as the OmpA and Pal proteins from *Acinetobacter baumannii*, and the PompB protein from *Vibrio alginolyticus* (Fig. 4). At the structural level, the residues superimpose and the interaction network is identical, although the structure of PompB contains no ligand in the pocket (Fig. 5).

## Discussion and Conclusion

Secretion systems in Gram-negative bacteria are large multiprotein complexes comprising proteins from the IM and OM. In this study we focus on the last discovered type IX secretion system (T9SS), a complex translocon only found in species of the *Bacteroidetes* phylum. To fit the specific needs of each bacterium, T9SSs have evolved to secrete various substrates such as toxins involved in pathogenesis, S-layer to confer protection, or adhesins for gliding motility. A number of researches have shown that more than 14 proteins are involved in this still ambiguous process, with some playing roles in the post-translational modification of T9SS substrate proteins^9^.

Heath *et al*.^17^ have shown that *porE* inactivation in *P. gingivalis* caused a loss of cell-associated proteinase activity, while immunoblotting and proteomic analyses revealed that virulence factors precursor proteins accumulated in the periplasm and were not targeted to the membrane. This provided strong evidence that PorE is a component of the T9SS in *P. gingivalis* essential for the proper secretion of T9SS substrates. Furthermore, sequence analysis revealed that PorE is a lipoprotein anchored in the OM through N-terminal acylation, and is composed of four domains, from N to C-terminal: a tetratricopeptide repeats domain, a five bladed β-propeller domain, a predicted carboxypeptidase regulatory domain-like fold, and an OmpA_C-like domain. The C-terminal domain was predicted to be a peptidoglycan binding protein, suggesting that PorE could connect the OM and the peptidoglycan mesh.

The structure of the C-terminal domain of PorE reported here shows strong three-dimensional evolutionary relationship with known OmpA_C-like domains found in other bacteria. Moreover, a fragment of PG could be built into the electron density. This fragment, containing four residues of the stem peptide (_L_-Ala-γ-_D_-Glu-*meso*-DAP-_D_-Ala), penetrates deeply in the PG binding groove. Indeed, PG binding in PorE is very similar to that has already been described for other PG binding domain within the OmpA_C-like family. Thus our work brings experimental evidence that PorE interacts with the stem peptide that is present in the PG of *P. gingivalis*, which suggests that PorE could serve as a bridge between the OM and the PG network (Fig. 4). Interaction of PorE with native PG should be tested, as well as potential interaction with T9SS component(s) to confirm that PorE could anchor T9SS to the cell wall.

As previously pointed out, sequences homologous to PorE are present in 174 species of the *Bacteroidetes* phylum^17^. In many species, including *P. gingivalis*, PorE is unique. For instance, a search with the BLAST program in the genome of *Duncaniella muris*, a species highly abundant in the murine intestinal tract^38^ and that potentially possesses a functional T9SS as PorK-L-M-N homologs are present in its genome, results in one PorE homolog. Indeed, this protein shares the same architecture as PorE (46% sequence identity with 98% sequence coverage), it shares the three main residues involved in PG binding, suggesting that it actually interacts with PG, and it is predicted to be a lipoprotein. On the other hand, multiple PorE homologs are present in some species. For instance, fourteen PorE homologs are present in the genome of *Tannerella forsythia*, another periodontal pathogen^39^ that potentially possesses a functional T9SS (around 50% sequence identity with more than 95% sequence coverage, conservation of the PG binding residues, and prediction as lipoproteins). Noteworthy, more than twenty PorE homologs are present in the genome of *F. johnsoniae* (25-30% sequence identity with more than 95% sequence coverage, and conservation of the PG binding residues). However, while these proteins have a signal peptide, except one, suggesting that they are translocated in the periplasm as PorE, none is predicted to be a lipoprotein. Further investigations would be necessary to reveal whether one of these *F. johnsoniae* proteins could have a function in T9SS homologous to PorE. Nevertheless, this result highlights that anchoring of T9SS into the cell wall might be different in the two bacterial models used for T9SS study, *P. gingivalis* and *F. johnsoniae*.

## Methods

### Cloning, protein expression, purification and analysis

The full length PorE (PGN_1296; Gene accession (gi):188594840) and its four protein domains were cloned using the Restriction Free method^40^. The full length sequence of PorE except the signal peptide and the first cysteine proposed to be acylated (residues 25–672) was amplified from *P. gingivalis* ATCC3327 chromosomal DNA using primers 5′-CCGAGAACCTGTACTTCCAATCAAAGAGCGTGAAGTTGAAAGATGCGG and 5′- CGGAGCTCGAATTCGGATCCTTATTATTAACGCAACTCTTCTTCGATCAC. The sequences of PorE individual domains starting from the N-terminus to C-terminus: i) tetratricopeptide repeat domain (TPR, residues 25–149; primers 5′- CCGAGAACCTGTACTTCCAATCAAAGAGCGTGAAGTTGAAAGATGCGG and 5′-CGGAGCTCGAATTCGGATCCTTATTATTCCTTTTGCTGGCGGGCAAAGAG), ii) β–propeller domain (WD40, residues 167–435; primers 5′-CCGAGAACCTGTACTTCCAATCAGACTTCGGCCCGGCCTATGCACCC and 5′-CGGAGCTCGAATTCGGATCCTTATTACAGTTCGAAATGGAAGAGGTGCGG), iii) carboxypeptidase regulatory domain-like fold (CRD, residues 441–527; primers 5′-CCGAGAACCTGTACTTCCAATCAACCGAGATTCAAGGTTATGTGATGG and 5′-CGGAGCTCGAATTCGGATCCTTATTACGATGCAAGGAAAAAGTCCACATA), and iv) OmpA_C-like putative peptidoglycan-binding domain (residues 534–668; primers 5′-CCGAGAACCTGTACTTCCAATCATTGCAAAATATTTTCTATGATTTCGAT and 5′-CGGAGCTCGAATTCGGATCCTTATTATTAACGCAACTCTTCTTCGATCAC) were also amplified from *P. gingivalis* ATCC3327 chromosomal DNA. All constructs were cloned into the pLIC03 expression vector that is a pET-28a+ derivative (Novagen) carrying a cassette coding for a His_6_ tag and a Tobacco Etch Virus (TEV) protease-cleavage site followed by the suicide gene *sacB* flanked by *Bsa*I restriction sites downstream the start codon.

Protein expression was carried out as previously described^41^. *E. coli* T7 transformants were grown in TB medium at 37 °C until reaching an Optical Density at λ = 600 nm (OD_600nm_) of 0.6–0.8. The expression was induced by adding 1 mM IPTG and the bacterial growth was pursued for 18 h at 17 °C. The cells were harvested by centrifugation at 2,500 × *g* for 15 min at 6 °C and the dried pellet was stored at −80 °C. After thawing, the cell pellet was resuspended in lysis buffer (50 mM Tris-HCl pH 8.0, 300 mM NaCl, 10 mM imidazole, 250 µg.mL^−1^ lysozyme, 10 µg.mL^−1^ DNAse, 10 mM MgSO_4_ and 1 mM PMSF) and the cells were lysed by sonication on ice. The pellet and soluble fractions were separated by centrifugation (16 000 g for 30 min). The His_6_-tagged protein was purified from the soluble fraction by immobilized metal ion-affinity chromatography using a 5 ml HisTrap Crude (GE Healthcare) Ni2 + -chelating column equilibrated in buffer A (50 mM Tris-HCl pH 8.0, 300 mM NaCl, 10 mM imidazole). The protein was eluted with buffer A supplemented with 250 mM imidazole and was further purified by size-exclusion chromatography (HiLoad 16/60 Superdex 200 Prep Grade, GE Healthcare) equilibrated in 10 mM HEPES pH 7.4, 200 mM NaCl. For crystallization trials, the purified protein was concentrated by centrifugation to 35 mg.mL^−1^ using an Amicon 10 kDa cut-off concentrator.

For the Size-Exclusion Chromatography Multi-Angle Light Scattering (SEC-MALS) analysis, the purified, concentrated protein was loaded on a Superdex 200 Increase 10/300 GL column (GE Healthcare) equilibrated in 10 mM HEPES pH 7.4, 200 mM NaCl at a flow rate of 0.6 mL.min^−1^, using an Ultimate 3000 HPLC system (Fischer Scientific). Detection was performed using an eight-angle light-scattering detector (DAWN8, Wyatt Technology) and a differential refractometer (Optilab, Wyatt Technology).

Selenomethionine-labeled (SeMet) OmpA_C-like domain was produced in *E. coli* T7 cells cultured in SeMet minimal medium^42^ at 37 °C until reaching an OD_600nm_ of 0.6–0.8. The expression was induced by adding 1 mM IPTG and the bacterial growth was pursued for 18 h at 17 °C. Cell lysis and protein purification were performed with the same protocol as for native OmpA_C-like domain.

### Crystallization of the OmpA_C-like domain

Crystallization screens using the purified samples of native and SeMet OmpA_C-like proteins were performed by the sitting-drop vapour diffusion method at 293 K in 96-well Swissci-3 plates with Stura Footprint (Molecular Dimensions), Wizard I and II (Rigaku) and Structure I and II (Molecular Dimensions) screens. The reservoirs of the Swissci-3 plates were filled by a TECAN pipetting robot, and the nanodrops were dispensed by a Mosquito robot (TTP Labtech). Crystals of OmpA_C-like domain appeared in several conditions with polyethylene glycols and sodium ions. The diffraction quality crystals for both native and SeMet OmpA_C-like domains achieved the maximum size after 7 days by mixing the protein solution with 0.1 M sodium acetate, 0.1 M MES pH6.5, and 30% (w/v) PEG 2000. Crystals were mounted in cryo-loops (Hampton CrystalCap Magnetic) and were briefly soaked in crystallization solution supplemented with 20%(v/v) ethylene glycol. The crystals were flash-cooled in a nitrogen-gas stream at 100 K using a home cryocooling device (Oxford Cryosystems).

### Data collection and processing, and Accession Code

X-ray diffraction data were collected at Soleil synchrotron Proxima1 beamline and were processed using XDS package^43^. The structure of the OmpA_C-like domain was determined by single wavelength anomalous diffraction (SAD) using data collected at the selenium X-ray absorption peak (0.97911 Å) from a selenomethione-derived crystal. The substructure determination for four methionine sites and the phase calculation were performed in Phaser^44^ as embedded in Phenix^45^. Model building was performed by the multivariate algorithm in Crank2 that simultaneously combines the structure determination steps, including the scaling and extracting the anomalous signal, finding the heavy atom sites, and solvent flattening^46^. The initial structure model was improved through iterative refinement with AutoBuster^47^ and manual refitting with COOT^48^. The validity of the refined structure was assessed by MolProbity^49^. Data collection and refinement statistics of the PorE OmpA_C-like domain are reported in Table 1. The atomic coordinates and structure factors have been deposited in the Protein Data Bank (PDB) under accession code 6TOP.

## Supplementary information

Supplementary information.
