# Author Correction: Crystal structure of Type IX secretion system PorE C-terminal domain from *Porphyromonas gingivalis* in complex with a peptidoglycan fragment

**DOI:** 10.1038/s41598-021-82414-x

**Published:** 2021-01-27

**Authors:** Nhung Thi Trang Trinh, Hieu Quang Tran, Quyen Van Dong, Christian Cambillau, Alain Roussel, Philippe Leone

**Affiliations:** 1 Architecture et Fonction des Macromolécules Biologiques, Aix-Marseille Université, UMR 7257, 163 Avenue de Luminy, Case 932, 13009 Marseille, France; 2 Architecture et Fonction des Macromolécules Biologiques, Centre National de la Recherche Scientifique, UMR 7257, 163 Avenue de Luminy, Case 932, 13009 Marseille, France; 3 Institute of Biotechnology, Vietnam Academy of Science and Technology, 18 Hoang Quoc Viet, Ha Noi, Vietnam; 4 University of Science and Technology of Hanoi, Vietnam Academy of Science and Technology, 18 Hoang Quoc Viet, Ha Noi, Vietnam; 5 Faculty of Medical Technology, PHENIKAA University, Yen Nghia, Ha Dong, Hanoi, 12116 Vietnam; 6 PHENIKAA Research and Technology Institute (PRATI), A&A Green Phoenix Group JSC, No. 167 Hoang Ngan, Trung Hoa, Cau Giay, Hanoi, 11313 Vietnam

Correction to: *Scientific Reports* https://doi.org/10.1038/s41598-020-64115-z, published online 30 April 2020

In the original version of this Article, Nhung Thi Trang Trinh was incorrectly affiliated with “Institute of Biotechnology, Vietnam Academy of Science and Technology, 18 Hoang Quoc Viet, Ha Noi, Vietnam” and “University of Science and Technology of Hanoi, Vietnam Academy of Science and Technology, 18 Hoang Quoc Viet, Ha Noi, Vietnam”.

The correct affiliations for Nhung Thi Trang Trinh are listed below.

Architecture et Fonction des Macromolécules Biologiques, Aix-Marseille Université, UMR 7257, 163 Avenue de Luminy, Case 932, 13009, Marseille, France.

Architecture et Fonction des Macromolécules Biologiques, Centre National de la Recherche Scientifique, UMR 7257, 163 Avenue de Luminy, Case 932, 13009, Marseille, France.

Faculty of Medical Technology, PHENIKAA University, Yen Nghia, Ha Dong, Hanoi 12116, Vietnam.

PHENIKAA Research and Technology Institute (PRATI), A&A Green Phoenix Group JSC, No. 167 Hoang Ngan, Trung Hoa, Cau Giay, Hanoi 11313, VietNam.

This has now been corrected in the PDF and HTML versions of the Article.

