# Supplementary material for: Crystal structure of Type IX secretion system PorE C-terminal domain from Porphyromonas gingivalis in complex with a peptidoglycan fragment
Source: Sci Rep. 2020 Apr 30;10:7384. doi: 10.1038/s41598-020-64115-z (PMC7192894; doi:10.1038/s41598-020-64115-z)

**Supplementary information**

**Crystal structure of Type IX secretion system PorE C-terminal domain from *Porphyromonas gingivalis* in complex with a peptidoglycan fragment.**

Nhung Thi Trang Trinh, Hieu Quang Tran, Quyen Van Dong, Christian Cambillau, Alain Roussel and Philippe Leone

**Supplementary Figure 1 :** Size-Exclusion Chromatography Multi-Angle Light Scattering (SEC-MALS) analysis of the PorE OmpA_C-like domain.

The UV absorbance chromatogram and the measured molar mass (17.7kDa) are shown in blue and red, respectively. The figure was prepared using MICROSOFT Excel (version 16.16.20).


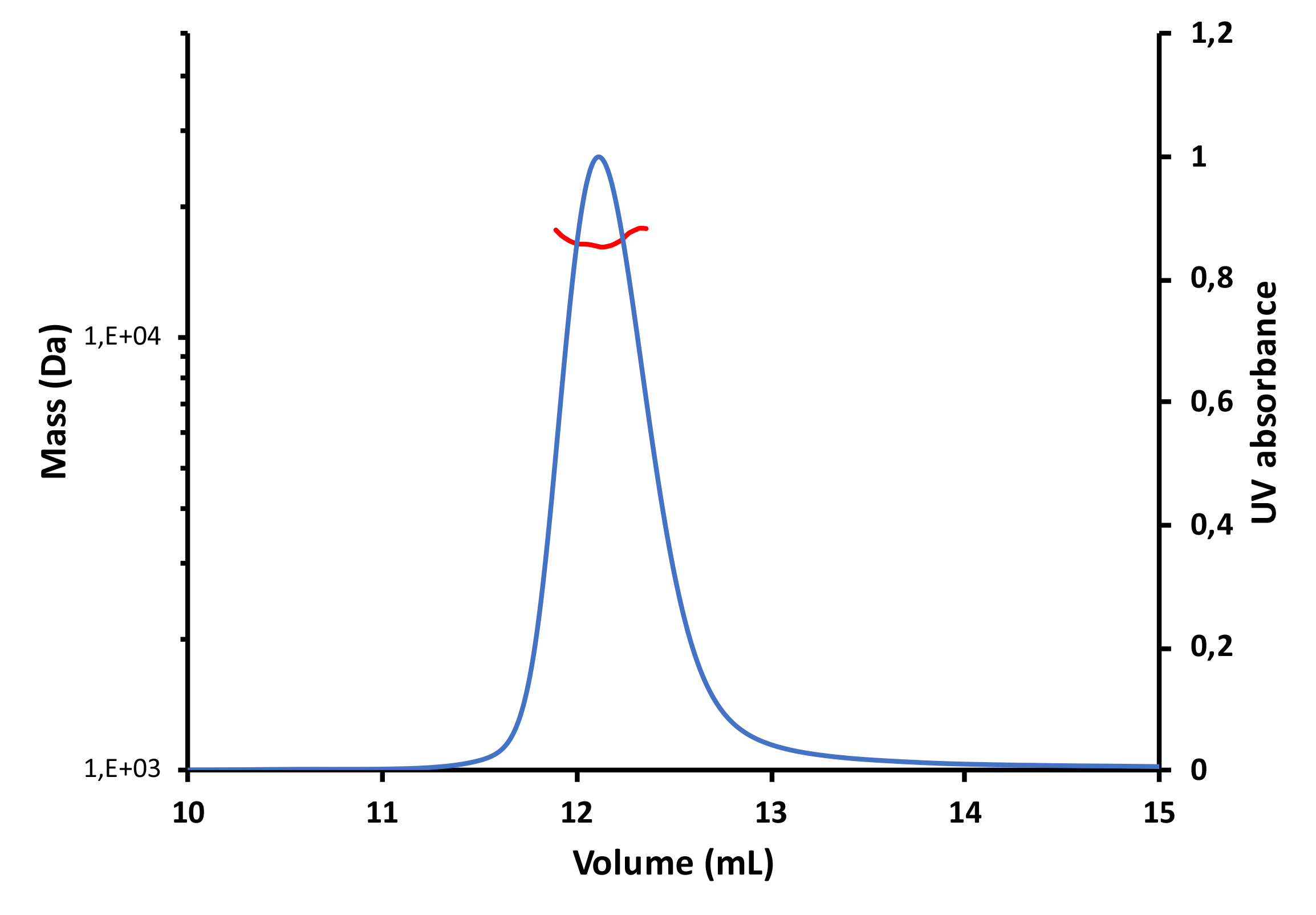

Supplement: Supplementary file 1 — Supplementary information. [file 41598_2020_64115_MOESM1_ESM.docx]
